# Physiological changes in response to apnea impact the timing of motor representations: a preliminary study

**DOI:** 10.1186/1744-9081-10-15

**Published:** 2014-04-28

**Authors:** Franck Di Rienzo, Nady Hoyek, Christian Collet, Aymeric Guillot

**Affiliations:** 1 Centre de Recherche et d’Innovation sur le Sport, EA 647, Université de Lyon, Université Claude Bernard, Lyon 1, Performance Motrice, Mentale et du Matériel (P3M), 27-29 Boulevard du 11 Novembre 1918, Villeurbanne, Cedex 69622, France; 2 Institut Universitaire de France, 103 boulevard Saint-Michel, Paris 75005, France

**Keywords:** Mental chronometry, Motor imagery, Apnea, Heart rate, Internal clock

## Abstract

**Background:**

Reduced physiological arousal in response to breath-holding affects internal clock processes, leading swimmers to underestimate the time spent under apnea. We investigated whether reduced physiological arousal during static apnea was likely to affect the temporal organization of motor imagery (MI).

**Methods:**

Fourteen inter-regional to national breath-holding athletes mentally and physically performed two 15 m swimming tasks of identical durations. They performed the two sequences in a counterbalanced order, the first while breathing normally using a scuba, the second under apnea. We assessed MI duration immediately after completion of the corresponding task. Athletes performed MI with and without holding breath.

**Results:**

MI durations (26.1 s ± 8.22) were significantly shorter than actual durations (29.7 s ± 7.6) without holding breath. Apnea increased MI durations by 10% (± 5%). Heart rate decrease in response to breath-holding correlated with MI durations increase (p < .01). Under apnea, participants achieved temporal congruence between MI and PP only when performing MI of the apnea swimming task. Self-report data indicated greater ease when MI was performed in a physiological arousal state congruent with that of the corresponding motor task.

**Conclusions:**

Physiological arousal affected the durations of MI through its effects on internal clock processes and by impacting the congruency in physiological body states between overt and covert motor performance. Present findings have potential implications with regards to the possibility of preventing underestimation of durations spent under a state of reduced physiological arousal.

## Introduction

Motor Imagery (MI) is the mental representation of a motor act without engaging in its actual execution. A large body of neuroscience research supports that MI and physical practice (PP) of the same task share overlapping neural substrates [1-4], and involve functionally equivalent neural processes [5-8]. As well, behavioral research provided strong evidence for a temporal congruence between the temporal structure of MI and the temporal parameters of the corresponding motor performance, reflecting the involvement of common planning and programming strategies [9]. Recently, Guillot et al. [10] reviewed the key-components that may affect the durations of MI, *i.e.*, factors that promote temporal congruence or conversely yield to consistent overestimation or underestimation of PP durations. Interestingly, they specifically highlighted the selective influence of several external factors on MI durations. For instance, the characteristics of the motor skill are likely to alter the temporal congruence between MI and PP durations (*i.e.*, short-lasting motor sequences are usually overestimated, whereas long-lasting and cyclical motor skills tend to be underestimated). Similarly, complex motor sequences require greater time to be mentally rehearsed compared to physically executed [9,11,12]. Finally, a proportional increase in MI durations was found along with the intensity of the mental effort [13-15].

While some intrinsic features of the task might impact its imagined duration, the sport imagery literature acknowledges that the construction of the temporal organization of MI is primarily based on the temporal parameters of the corresponding motor performance [10,16,17]. Indeed, actual motor experiences participate to the central elaboration of forward models [18], *i.e.*, an internal simulation of the movement derived from the efferent copy of the motor command, predicting future body states and stimulating the sensory consequences of the expected actions [18-21]. Accordingly, central processing of MI involves the forward modeling of the corresponding action, from which substantial information about its spatial and temporal features is obtained [18,21-23]. Albeit primarily showed in chronometric experiments, this claim is also supported by brain imaging data. For instance, Lorey et al. [24] stated that:

*“The functional equivalence of action states is due mostly to internal estimations of the expected sensory feedback in both motor control and motor imagery”* (p. 1).

Also, applied experiments provided evidence that central integration of sensory input during PP affected MI durations, due to central updating of the temporal structure of the motor representation [15,22,25,26]. Di Rienzo et al. [26] observed that physical fatigue led to underestimation of the durations of imagined turns in swimming athletes. A tight temporal congruence between MI and PP was measured before fatigue state, but MI durations were shorter immediately after an intense swimming exercise. This was only observed for MI tasks that involved a strong reference to bodily information (*i.e.*, first-person visual MI) [27]. Conversely, external visual imagery durations were unaffected by physical fatigue. The authors interpreted their results as an account of the update of the forward model due to the repeated integration of sensory feedbacks under fatigue state ([see also [23]). However, findings might also account for aversive or motivational effects related to the experience of physical fatigue. Finally, scientific data in clinical populations also support the hypothesis of sensory afferences playing a crucial role in the shaping of MI durations (*e.g.*, [28]).

While cognitive motor processes participate to the elaboration of the temporal structure of MI [29,30], Decety et al. [31] early stated that *“MI involve*[s] *the same clock mechanism as the actual motor behavior”*, with reference to Keele et al. [32]. The authors observed that overestimation of MI durations in stroke patients might account for “*an impairment in the central implementation of the clock*” (*i.e.*, in the sense of the internal clock) [33,34]. In other words, MI durations might not only depend on the temporal parameters of forward models but also from internal clock processes generally involved in time estimation ability. Since Hoagland [33], theoretical models for a human internal clock were conceptualized from animal research (for a review, see [35]). Internal clock models assume that temporal features are centrally represented on the basis of a physiological “*pacemaker*” producing pulses at a given rate (the lick rate was assumed to play the role of the pacemaker in rats, see [36]). The amount of pulses within a given time period would be summed, stored within working memory, and finally compared with a reference long-term memory model (for an overview, see [34,37,38]). Applied to motor control, Treisman et al. [39] provided evidence that an oscillator controlled temporal estimations in humans and was involved in the control of movement timing.

Interestingly, a large body of experimental data supports that heartbeats may be the physiological pacemaker of the internal clock in humans [38,40-43]. Namely, humans might non-consciously refer to their own heartbeats to achieve time estimations. This postulate accounts for the reported effects of physiological arousal on time estimation ability [33,41,44-47]. Albeit it was recently suggested that physiological arousal, but not heart rate *per se*, influenced time estimation in humans, thus putting into debate the concept of *“internal pacemaker” *[48], physiological arousal and heart rate remain highly correlated. Past experiments also demonstrated that physiological arousal affected MI durations (see [10] for a review). For instance, Louis et al. [49] observed that reduced body arousal elicited by relaxation resulted in longer MI durations compared to both aroused and baseline conditions. In the same vein, Gueugneau et al. [22] found that the duration of MI followed the circadian rhythms of arousal, *i.e.*, longer MI durations were measured early in the morning and late in the evening (see also [50]). To summarize, findings of a relationship between physiological arousal and MI durations are congruent with the working hypothesis of internal clock processes being involved in the temporal organization of MI. Nonetheless, the cognitive interplay between internal clock processes and time parameters of forward models remains at this point incompletely understood.

Voluntary breath-holding (*i.e.*, apnea) at rest is known to decrease heart rate both in air and water environments [38,43,51,52]. Bradycardia occurs in response to water immersion due to cardiovascular and metabolic changes triggered by the mammalian diving reflex [53-56]. In a pioneering study, Jamin et al. [43] observed that reduced physiological arousal during breath-holding caused an underestimation of the durations of static apneas by about 20%. According to the authors, bradycardia disturbed the internal clock and impaired time estimation ability. These findings are of specific concern for breath-holders, because accurate control of the time spent under apnea is crucial to achieve safe practice and avoid syncope accidents [38].

The present study used the unique model of apnea to understand the respective contribution of *i)* time parameters of forward models derived from *past* motor experiences and *ii)* internal clock processes depending on the *current* physiological body state on the temporal features of MI. We hypothesized that breath-holding might affect MI durations due to changes in the internal clock, and that these effects would be selective according to the arousal body state during PP.

## Methods

### Participants

Fourteen breath-holding athletes were requested to physically and mentally perform a set of two swimming tasks, the first with, and the second without simultaneously holding breath. The experimental group consisted of 14 breath-holding athletes (males = 11, females = 3; *M* = 38.3, *SD* = 7.7) and included both national (*n* = 9) and inter-regional (*n* = 5) athletes. The experimental design was approved by the Research Ethics Committee of the University. Informed written consent was obtained according to the principles and statements of the Declaration of Helsinki (1964).

### Experimental design and settings

**Figure 1:**
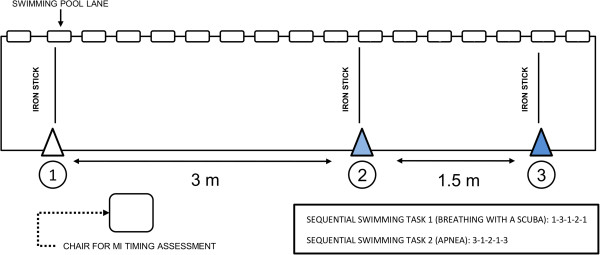
**Overhead view of experimental settings for the two sequential swimming tasks.** MI: Motor Imagery.

Experiments were individually implemented in a 25 m public swimming pool (F-69110, Sainte Foy-lès-Lyon). Water temperature was 27.3°C and participants wore an apnea suit and were not weighted. Three iron sticks were initially dived at the bottom of the pool, perpendicularly to the lane. Iron sticks were respectively separated from each other by 4.5, 3.0 and 1.5 m (Figure 1). All experimental sessions were carried out in the same daytime (8 pm), *i.e.*, when MI performance is the most accurate [22,50].

### Swimming tasks

Participants were requested to physically and mentally perform two swimming tasks of 15 m involving a sequence of aquatic displacements between 3 iron stick positions. The first task was a 1-3-1-2-1 sequence and the second was a 3-1-2-1-3 sequence (Figure 1). The first swimming task was performed while breathing normally using a scuba after a 45 s period of rest where participants breathed normally behind the starting iron stick (PP_BREATHE_). The second task was performed under apnea following a 45 s period of static apnea behind the starting iron stick (PP_APNEA_). Pilot recordings showed that this duration was sufficient to elicit a sustained bradycardia without eliciting any discomfort for participants. The two tasks were of equivalent difficulty and involved a similar number of turns. During both tasks, participants were instructed to swim using self-paced breaststroke actions, and to remain by the surface of the pool (*i.e.*, a part of their body had to be constantly visible out of water surface by the experimenter). Concerning the turns, after the head had passed the iron stick, participants turned themselves and swam in the reverse direction. PP_BREATHE_ and PP_APNEA_ were presented in a randomized order across participants. Four PP trials were completed before measurements of MI durations.

The timekeeping of PP_BREATHE_ and PP_APNEA_ was manually performed by a professional swimming coach who was not informed of the purpose of the study. Timekeeping was triggered when breath-holder’s head passed above the starting and ending iron sticks (*i.e.*, *1*-3-1-2-*1* for PP_BREATHE_ and *3*-1-2-1-*3* for PP_APNEA_) (Figure 1).

### Motor imagery ability assessment

Breath-holding participants completed, before the experiment, a validated MI questionnaire to assess their ability to engage into MI (MIQ-3 questionnaire) [57]. The MIQ-3 consists in 12 items for which participants rate the ease/difficulty encountered during MI on 7-point scales (ranging from 1: *“very hard to see/feel”* to 7: *“very easy to see/feel”*) referring to external visual, internal visual, and kinesthetic MI.

Mental chronometry allows investigation of the time course of information processing within the central nervous system [58]. We measured MI duration immediately after PP of the corresponding motor task. Participants sat comfortably in a chair placed by the side of the swimming pool (Figure 1). Participants were instructed to perform MI of the sequence using first-person visual and kinesthetic MI concurrently. For PP_BREATHE_ and PP_APNEA_, they performed MI while breathing normally after a 45 s period of normal breathing (MI_BREATHE_) and while holding breath after a 45 s period of static apnea (MI_APNEA_). The two MI conditions were presented in a randomized order (3 trials each). Participants orally indicated the onset and end of each MI trial.

After completion of MI_BREATHE_ and MI_APNEA_, participants reported their level of MI ease/difficulty on a 10-point Lickert scale (ranging from 1: *“very easy”* to 10: *“very hard”*).

### Cardiac activity

We recorded the cardiac activity using a heart rate monitor (Polar® RCX3, France) providing the instantaneous heart rate (IHR) in beats per minute (bpm). HR at rest (HR_REST_) was individually evaluated before the onset of experiments. HR_REST_ was considered the average value obtained during 45 s of rest during which participants remained motionless, comfortably seated on the chair by the side of the pool (Figure 1).

Voluntary apnea with face immersion in humans is commonly associated with anticipatory tachycardia. Cardiac activity starts to decrease and stabilizes after approximately 30 s of face immersion (*e.g.*, [59]). We could not monitor cardiac activity during the swimming tasks, which required a *waterproof* heart rate monitor. Yet, bradycardia in response to breath holding is largely documented in the literature, and is particularly marked due to the mammalian diving reflex [51,53,56,60].

We collected the average HR during the 45 s period preceding MI and expressed it in percentage of HR_REST_ for normalization across participants. We did not collect HR data during MI due to solid literature background reporting MI-related changes in cardiac activity (*i.e.*, cardiac responses during MI closely mirror physiological changes elicited by PP of the same task and/or recruitment of attentional resources) [61-64].

### Statistical analyses

All statistical calculations were performed using the R freeware [65], and the alpha threshold for significance was settled at α = 5%.

MIQ-3 scores represent discrete variables derived from ordinal scales. Due to assumption of non-Gaussian distribution, they were compared across internal visual, external visual and kinesthetic MI modalities using Friedman’s test. Self-reports of ease/difficulty were compared between MI_BREATHE_ and MI_APNEA_ using paired Wilcoxon’s tests.

Normality of chronometric data was checked through visual inspection of Q-Q plots. PP, MI_BREATHE_ and MI_APNEA_ durations were compared for both swimming tasks using a repeated measures MANOVA model with the swimming task as group factor (PP_BREATHE_ and PP_APNEA_) and the task type as repetition factor (PP, MI_BREATHE_ and MI_APNEA_). An independent analysis of PP, MI_BREATHE_ and MI_APNEA_ durations was conducted for PP_BREATHE_ and PP_APNEA_ using one-way ANOVAs with repeated measures. We used paired Student’s t-tests with Holm’s Bonferroni corrections (Holm, 1969) for post-hoc comparisons.

For both swimming tasks, independence between HR and MI duration changes was tested using Pearson’s product-moment correlation between the percentage of HR change before MI_BREATHE_ and MI_APNEA_ and the percentage of change in MI_BREATHE_ and MI_APNEA_ durations.

## Results

### MIQ-3 scores

Testing the MI modality effect on MIQ-3 scores did not reveal statistically significant differences (Friedman’s *Chi-squared* = 1.45, *p* = .48). Namely, breath-holders reported similar degrees of difficulty at performing external visual (*M* = 5.84, *SD* = .69), internal visual (*M* = 6.0, *SD* = .80) and kinesthetic MI (*M* = 5.80, *SD* = .97). Mean MIQ-3 scores (*M* = 5.80, *SD* = .59) also showed that participants experienced MIQ-3 tasks as easy to visualize or perceive.

### Likert ratings

Breath holders self-reported greater MI ease scores during MI_BREATHE_ (*M* = 3.21, *SD* = .65) as compared to MI_APNEA_ (*M* = 4.93, *SD* = .70) when imagining the PP_BREATHE_ task (Wilcoxon’s *W* = 3.5, *p* = .005). Conversely, lower ratings were obtained during MI_APNEA_ (*M* = 3.58, *SD* = .62) compared to MI_BREATHE_ (*M* = 5.15, *SD* = .52) when imagining the PP_APNEA_ swimming task (Wilcoxon’s *W* = 44, *p* = .01).

### Temporal data

MANOVA with repeated measures yielded the main effect of Task (*F*_*(2,25)*_ = 10.16, *p* < .001, *η*^*2*^ = .44) while there was a trend concerning the interaction between the swimming task and task type (*F*_*(2,25)*_ = 2.96, *p* = .06, *η*^*2*^ = .12).

**Figure 2:**
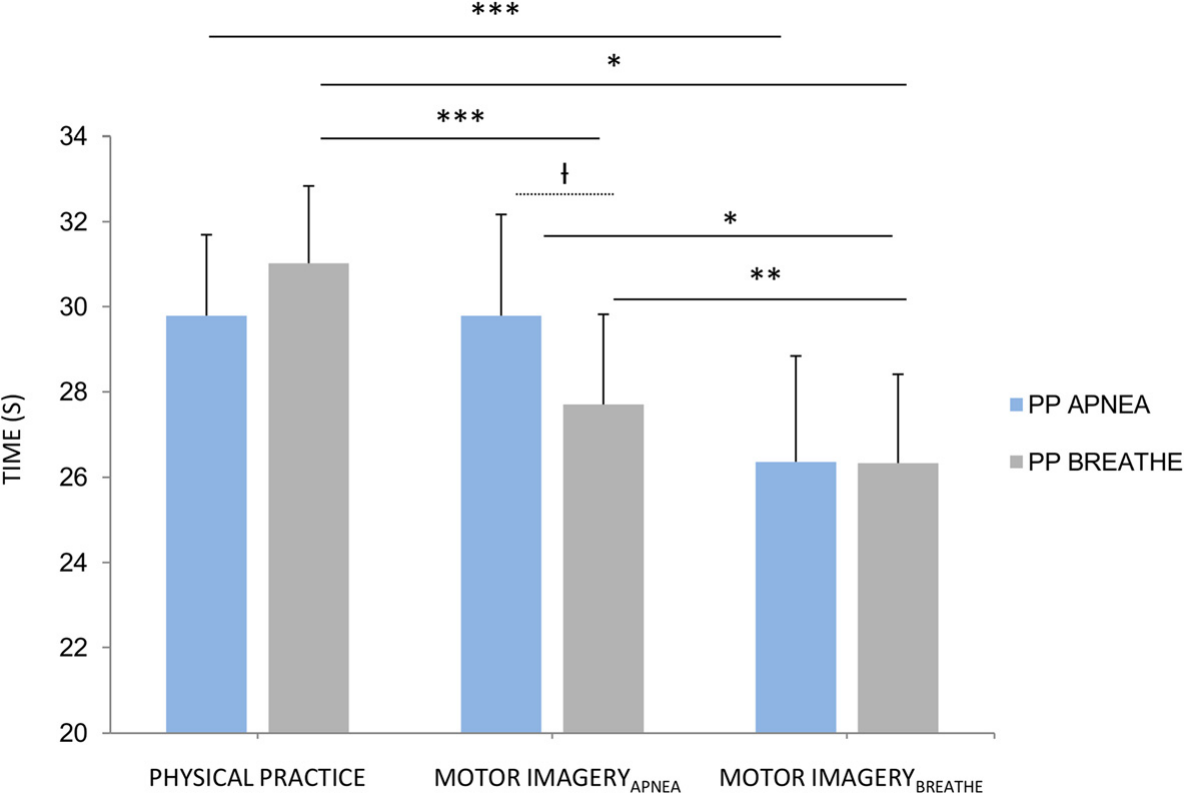
**Physical practice and motor imagery durations during breathing and apnea swimming tasks.** Error bars represent mean standard error. Only significant differences were graphically represented. PP: Physical Practice. Ɨ: p < .10, *: p < .05, **: p < .01, ***: p < .001.

ANOVA with repeated measures revealed that the effect of task type was statistically significant for both PP_BREATHE_ and PP_APNEA_ (*F*_*(2,26)*_ = 4.19, *p* = .02 and *F*_*(2,26)*_ = 20.44, *p* < *.001*, respectively). For both swimming tasks, post-hoc analyses revealed higher durations during PP compared to MI_BREATHE_ (*p* = .02 and *p <* .001, respectively), as well as during MI_APNEA_ compared to MI_BREATHE_ (*p* = .02) as shown by Figure 2. Post-hoc analyses also revealed shorter durations during MI_APNEA_ (*M* = 27.71, *SD* = 8.18) compared to PP_BREATHE_ (*M* = 31.02, *SD* = 7.15; *p* < .001), whereas no difference emerged between MI_APNEA_ (*M* = 29.78, *SD* = 9.95) and PP_APNEA_ (*M* = 29.79, *SD* = 6.73) (*p* = .99). As there was no difference between PP_BREATHE_ and PP_APNEA_ durations (*p* = .72), the patterns of MI *vs.* PP differences in durations across swimming tasks might account for the trend in the MANOVA model, albeit the difference between the two MI_APNEA_ durations was only marginally significant when corrections for multiple comparisons were applied (*M* = 2.07 s, *SD* = 3.47, *p* = .09), see Figure 2. Finally, the difference in durations between MI_BREATHE_ of the PP_BREATHE_ and PP_APNEA_ tasks was far from significance (*p* = .98) as shown by Figure 1.

### Cardiac activity

HR data were processed from 13 participants as data from one participant was lost due to a technical failure. Overall, average HR before MI_BREATHE_ was 72.61 bpm (*SD* = 2.29) while average HR before MI_APNEA_ was 66.81 bpm (*SD* = 2.24). Significant differences were observed in HR before the onset of the two MI conditions for both PP_BREATHE_ (7.0% of HR_REST_ decrease at onset of MI_APNEA_ as compared to MI_BREATHE_; *t* = 3.91, *μ* = 0, *p* = .002) and PP_APNEA_ (9.0% of HR_REST_ decrease at onset of MI_APNEA_ as compared to MI_BREATHE_; t = 4.27, *μ* = 0, p = .001).

**Figure 3:**
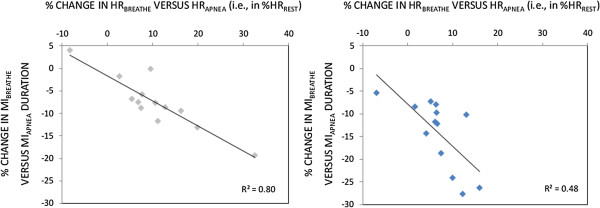
**Correlations between variations in heart rate (percentage from basal values) and motor imagery duration changes between normal breathing and apnea conditions, for the apnea (left panel) and breathing (right panel) swimming tasks.** MI: Motor imagery, HR: Heart rate.

A correlation between the percentage of HR decrease between periods preceding MI_BREATHE_ and MI_APNEA_ and the percentages of MI durations increase between MI_BREATHE_ and MI_APNEA_ was found for both PP_BREATHE_ and PP_APNEA_ (*R*^*2*^ = .80, *p* < .001 and *R*^*2*^ = .48, *p* < .01, respectively). The sum square error of the regression was 66% higher for PP_BREATHE_ compared to PP_APNEA_ (Figure 3).

## Discussion

The present study was designed to investigate the effect of apnea on MI duration, and understand the respective contribution of the temporal parameters of both forward models and internal clock processes to the elaboration of the temporal structure of MI. We assessed participants ability to engage into MI using a validated MI questionnaire [57]. We recorded the durations of two swimming tasks performed with and without holding breath. For each task, we combined chronometric and physiological recordings of MI performed with and without holding breath.

MIQ-3 scores were similar between external visual, internal visual and kinesthetic imagery modalities. We specifically instructed participants to engage into first-person and kinesthetic MI concurrently as these modalities involve greater reference to one’s procedural memory [26,27]. No significant difference was found between the durations of the two swimming tasks. Both were physically performed using a similar swimming technique, involved the same number of turns and a similar distance. Interestingly, PP_BREATHE_ durations were underestimated during MI_BREATHE_. This result might account for the characteristics of the swimming sequence. Indeed, PP of both sequences lasted about 30 s. As recently reviewed by Guillot et al. [10], motor sequences longer than 15-20 s tend to be underestimated (*e.g.*, [17,66-68]). The reasons supporting this effect are not straightforward. Temporal parameters of movements may be quantitatively more complex to reproduce accurately in the case of long motor sequences, particularly for actions requiring an important amount of feedback control with reference to the action/control model by Glover [69]:

*“(…) the control of nonballistic actions is achieved by continuously regulating the movement during its execution so that the desired end state is achieved at the desired time. The lack of this information in imagery may be what causes a shortening of the duration of the imagined event” *[66], p. 880.

Due to the solid literature background supporting that motor experiences shape the temporal organization of MI [22,23,25,28], similar imagined durations were expected between the two swimming tasks. However, MI_APNEA_ exceeded MI_BREATHE_ durations regardless the swimming task involved. Performing MI while holding breath overall increased MI durations by 2.7 s (≈ 10%). Importantly, increased MI durations under the condition of breath-holding were correlated to the preceding HR decrease. This relationship was observed for both swimming tasks (Figure 3). These findings support the involvement of internal clock processes in the shaping of MI duration, as early postulated by Decety et al. [31]. As time perception and movement timing both involve reference to a common physiological oscillator [36,39], it is suggested that bradycardia affected the internal clock *“pacemaker” *[34,35,38,40-42], thus slowing the processing rate of motor representations. The involvement of the internal clock in the generation of MI durations brings new insights for a unified interpretation of experimental results related to factors affecting MI durations, such as the circadian modulation [22,50] or relaxed/aroused body states [49]. Interestingly, these studies reported increased MI durations under low physiological arousal and shortened MI durations under high physiological arousal. These changes were typically associated with different cardiac activities [26,49].

MI_APNEA_ durations remained shorter than that of PP for the swimming task involving normal breathing, whereas isochrony was found when comparing MI_APNEA_ and apnea swimming task durations. Longer durations (+2.07 s) were recorded during MI_APNEA_ of the apnea swimming tasks *vs.* MI_APNEA_ of the swimming task performed while breathing with a scuba. Yet, this difference was only marginally significant when corrections for multiple comparisons were applied (Figure 2). This unexpected finding suggests that congruence in physiological body states between overt and covert motor performance also contributes to the accuracy of MI durations. A limitation to this interpretation is that we did not record isochrony between MI_BREATHE_ and PP_BREATHE_. This mismatch was attributed to the task duration, thought to elicit an incomplete encoding of the temporal parameters [10,66]. The apnea swimming task was of identical duration but, crucially, was performed under a state of reduced physiological arousal. As movement timing parameters are controlled through reference to the internal clock [39], retention of movement timing parameters may be facilitated under the condition of reduced physiological arousal eliciting a slowing down of the internal clock [38,43]. While the underestimation of MI_BREATHE_ durations might account for the task duration in the case of normal breathing, it may rather relate to the physiological arousal mismatch during MI_BREATHE_ of the apnea swimming task. Albeit this interpretation remains an inference, forward models relate to current body states through integration of proprioceptive afferences [27,70]. For instance, the physiological body state affects the body representation through subtle changes in proprioceptive inputs to the central nervous system (see [71,72] for examples). Therefore, the arousal state is an intrinsic component of the central processing of the expected consequences of imagined actions (forward modeling). Overall, while reference to internal clock affected the processing rate of motor representations, accurate movement timing processing through forward modeling presumably required a state of congruence in physiological body states. The fact that the R-squared of the regression between changes in HR and the associated changes in MI durations was higher (66%) for the breathing compared to the non-breathing swimming task partly supports to this assumption, as in the latter case online changes in the internal clock accounted to a lesser extent for the associated changes in MI durations.

Isochrony between MI_APNEA_ and PP_APNEA_ may thus result from both efficient storage of timing parameters of actual movement, facilitated by the changes in the internal clock in response to apnea, and accurate processing of the expected sensory consequences of the imagined action due to congruence in physiological body states. Such assumption is congruent with the subjective reports. Breath holders reported greater MI ease when the physiological arousal condition before initiation of MI was similar to that preceding PP of the same task (*i.e.*, namely with or without holding breath during the 45 s preceding initiation of MI or PP). These preliminary results support the co-contribution of forward modeling and internal clock processes to the shaping of MI duration. Yet, this preliminary study must be extended by future designs involving different levels of arousal (*e.g.*, increased heart rate) and retention conditions (*e.g.*, performing MI two days later). Complementary measurement of physiological arousal are also necessary (*e.g.*, autonomic nervous system recordings), for instance under the conditions of MI and apnea underwater [73,74]. Another interesting issue would be determining to which extent changes in cerebral blood flow due to apnea might affect MI ability. For instance, accurate MI duration was associated with higher activation intensities within regions of the brain motor system [75]. As previous work underlined that apnea led to the underestimation of PP duration [38,43], future studies should seek to determine whether correcting the effect of breath holding on MI durations (*i.e.,* involving biased temporal predictions related to bradycardia amplitude), for instance by providing feedback concerning respect of isochrony with PP after completion of MI, would improve time estimation accuracy. Taken together, present findings might contribute to promote safe apnea practice in the currently developing context of apnea competitions.

## Competing interests

The authors declare no competing interests.

## Authors’ contributions

All authors (FDR, NH, CC and AG) participated to the design of the study. FDR and NH supervised the experiments. FDR performed the statistical analyses and drafted the manuscript. All authors read, amended and approved the final manuscript.
